# Cell-free DNA release by mouse placental explants

**DOI:** 10.1371/journal.pone.0178845

**Published:** 2017-06-16

**Authors:** Mark Phillippe, Sharareh Adeli

**Affiliations:** Vincent Center for Reproductive Biology, Department of Obstetrics & Gynecology, Massachusetts General Hospital, Boston, MA; Otto von Guericke Universitat Magdeburg, GERMANY

## Abstract

Although suggested that “fetal” cell-free DNA (cfDNA) is derived from trophoblast cells, the exact origin is unclear. The studies in this report sought to demonstrate that placental tissue releases cfDNA in parallel with cell death, that the size range of cfDNA is similar to that found in maternal plasma, and that the cfDNA fragments are able to stimulate a proinflammatory cytokine response. Placentas were harvested from near term pregnant CD-1 mice and cultured in DMEM/Ham’s F12/FBS media in 8% or 21% O_2_. After centrifugation to remove cells and cellular debris, the cfDNA was extracted from the media and quantified by DNA spectrophotometry. The cfDNA fragments were sized using a 1.5% TAE gel. Cell death was quantified by lactate dehydrogenase assay; and tissue homogenates were used to quantify caspase activity and BAX expression. Cultured RAW-264.7 macrophage cells were used to determine IL6 stimulation by cfDNA. The cfDNA levels released in 8% O_2_ (placental normoxia) were not significantly different from explants cultured in 21% O_2_ (placental hyperoxia). The cfDNA fragments ranged in size from < 100 –< 400 bp. The cfDNA release increased when cultured with LPS, whereas it decreased with trolox (vitamin E analog). Explant release of cfDNA increased in parallel with cell death. The cfDNA release and cell death of trophoblast appears to involve components of the apoptosis signaling pathway as suggested by LPS enhancement of placental caspase activity, suppression of cfDNA release by a pan-caspase inhibitor and the trend toward increased Bax protein expression. Studies with cultured macrophage cells confirmed the ability of cfDNA to stimulate an IL6 response. In summary, these studies have confirmed the ability of placental tissue to release significant amounts of cfDNA, a phenomenon that appears to be mediated, at least in part, by apoptosis; and that the cfDNA released by the placental explants is able to stimulate a significant proinflammatory response. Thus, these studies provide support for the hypothesis that cell-free fetal DNA released by placental tissue potentially plays a mechanistically important role during the events leading to the onset of parturition.

## Introduction

Although it has been suggested that cell-free “fetal” DNA is derived from trophoblast cells as they undergo normal turnover during cellular apoptosis and/or necrosis [1, 2], the exact origin for fetal DNA in maternal plasma during pregnancy is unclear. Zhong et al. [3] compared the concentration of cell-free fetal DNA to the number of fetal erythroblasts circulating in maternal plasma; and finding no correlation, these investigators concluded that the fetal DNA was derived from other sources, such as the placenta. Suggesting a possible placental origin for the fetal DNA found in maternal plasma, Goswami et al. [4] described increasing quantities of microparticles derived from apoptotic syncytiotrophoblast cells circulating in the maternal blood as pregnancy progressed to term. Bischoff et al. [2] utilized electron microscopy to demonstrate nucleosomal DNA fragments circulating in the plasma of pregnant women associated with the membrane fragments and apoptotic bodies. Reddy et al. [5] observed higher levels of syncytiotrophoblast microparticles and cell-free fetal DNA in maternal circulation at term in the plasma of women about to go into labor compared to samples collected from women not in labor. In an effort to demonstrate cell-free fetal DNA release by placental tissue, Tjoa et al. [6] utilized short-term human placental explant cultures to observe the release of cell-free DNA in association with oxidative stress and apoptosis.

The possibility that cell-free fetal DNA released by the placenta and/or fetal membranes serves as a trigger for parturition through its ability to stimulate an innate immune response resulting in the intrauterine production of proinflammatory cytokines and chemokines leading to cervical ripening, rupture of the membranes and the onset of phasic myometrial contractions has recently been proposed [7, 8]. The studies described in the present report sought further clarify the origin of cell-free fetal DNA through the use of mouse placental tissue explants, thereby allowing the direct observation of cell-free DNA (cfDNA) release by this tissue. In addition, these studies sought to determine whether placental tissue releases cfDNA in parallel with cell death (i.e. apoptosis and/or necroptosis), whether the size range of cfDNA released by the placental explants is similar to that of cell-free fetal DNA found in maternal plasma, and whether the cfDNA fragments released by the placenta are able to stimulate a proinflammatory response by macrophage cells.

## Materials and methods

### Placental explants

This study was carried out in strict accordance with the recommendations in the Guideline for the Care and Use of Laboratory Animals of the National Institutes of Health; the research protocol was approved by the Institutional Animal Care and Use Committee–Office of Laboratory Animal Welfare (Assurance # A3596-01) at the Massachusetts General Hospital, Boston MA. Placentas were collected from pregnant CD-1 mice on gestational days 15–18 using sterile surgical techniques. Timed-pregnant mice were purchased from Charles River Laboratories. The tissue harvests were performed under 3% isoflurane anesthesia; subsequently, the mice were euthanized by exsanguination while still under anesthesia. After rinsing with sterile phosphate buffered saline, the placentas were placed in sterile tissue culture media (45% DMEM/45% Ham’s F12/10% FBS + pen/strep) and each placenta was cut into 4 pieces; and then all 4 pieces were placed into individual wells of 6-well plates containing 2 mL of the tissue culture media. For placental explant cultures performed at physiologic O_2_ concentrations for placenta (i.e. 8% O_2_ levels) [9], the harvested placentas were placed in culture media that had been pre-bubbled with a sterile gas mixture containing 8% O_2_, 5% CO_2_ and 87% N_2_. After cutting and placing in 6-well dishes, these placental explants were cultured in an incubation chamber containing the same low oxygen gas mixture at 37°C for up to 21 hours. For placental explants cultured in hyperoxic conditions (for placenta), the tissue explants were incubated in chambers containing 5% CO_2_ and 95% room air (i.e. ambient O_2_ levels of 21%).

### DNA isolation

The culture media and placental pieces were collected at times zero, 6 and 21 hours. To assess cell-free DNA (cfDNA) released by the placental explants, the collected media samples were centrifuged at 8,000 xg to remove any cells or cellular debris; and then with 300 μL aliquots of the supernatant, cfDNA was extracted using a genomic DNA extraction technique (High Pure PCR Template Prep kits (Roche Applied Science)). The concentration of the isolated DNA was determined using a NanoDrop spectrophotometer (Thermo Scientific); and this concentration was used to calculate the total DNA content in the 2 mL of culture media. The combined weights of the 4 placental pieces were then used to normalize the DNA content for the amount of placental tissue; i.e. nanograms (ng) of cfDNA per milligrams (mg) of placental tissue. To determine the size of the cfDNA fragments, aliquots of extracted cfDNA were run on 1.5% agarose / Tris-acetate-EDTA (TAE) gels. To visualize the DNA bands, the gels were preloaded with GelRed dye (Biotum Corp.) and transilluminated with UV light.

### Explant treatments

Placental explant cultures were performed with the addition of various reagents as follows: staurosporine (0.5–3 μM) or thapsigargin (0.5–5 μM) to assess the effects of these stimulators of apoptosis [10, 11]; hydrogen peroxide (H_2_O_2_; 10–1,000 μM) to assess the effects of this stimulator of oxidative stress [12]; and ascorbic acid (Vitamin C; 1–5 mM) or trolox (Vitamin E analog; 0.5–3 mM) to assess the effects of these anti-oxidant vitamins [6] (all from Sigma-Aldrich Chemical). Additional studies were performed using the pan-caspase inhibitor Q-VD-OPh (10–200 μM) to assess the role of the caspase signaling pathway [13], necrostatin-1 (10–25 μM) to assess the role of necroptosis [14, 15], and lipopolysaccharide (LPS, 50–500 ng/mL) or tumor necrosis factor-α (TNFα; 25–100 ng/mL) to assess the effects of these inflammatory mediators [16] (all from Sigma-Aldrich Chemical). The culture media was used to assess overall cell death with the Cytotox Lactate Dehydrogenase (LDH) assay (Promega Corp.). Homogenates of the placental explants were utilized to quantify caspase-3 and -7 activity using the Caspase Glo 3/7 Assay (Promega Corp.) and the expression of the pro-apoptotic Bax protein using a mouse Bax ELISA kit (MyBioSource).

### RAW macrophage stimulation

Intact vertebrate DNA is a poor TLR9 agonist because of the inhibitory effects of guanine-rich sequences present in the telomere regions [17]. In an effort to demonstrate the ability of cfDNA to stimulate a proinflammatory effect, studies needed to be performed using cfDNA depleted of the high molecular weight telomere fragments by gel-mediated size isolation, as we have recently reported [18]. Specifically, the isolated cfDNA from the placental explants was resolved on 1.5% agarose/TAE gels; subsequently, gel blocks containing the < 100 –< 400 bp size fragments were excised, and the cfDNA in the gel blocks was isolated using PureLink Quick Gel Extraction kits (Invitrogen Life Technologies). The cfDNA was then further purified and concentrated using the Genomic DNA Clean & Concentrator-25 kits (Zymo Research Corp.).

To obtain the placental DNA depleted of telomere sequences, genomic DNA was isolated from mouse placentas; and then restriction endonuclease (RE) digested with MseI and Cac8I (New England Biolabs) for 2 hours at 37°C. Of note, RE treatment digests genomic DNA regions, but not the telomere regions because the latter are devoid of recognition sites for classic REs [19]); therefore, the telomere regions retain their high molecular weight sizes (i.e. thousands of base pairs) and do not significantly migrate into the gels. The RE digested placental DNA was resolved on 1.5% agarose/TAE gels; and gel blocks containing ~ 200–700 bp size fragments were excised. The DNA in the gel blocks was isolated and concentrated as described above. The concentration and the quality (based on the 260/280 nm ratio) of the telomere-depleted cfDNA and RE-digested genomic DNA isolates were determined using a NanoDrop spectrophotometer (Thermo Scientific, Wilmington, DE).

The *in-vitro* DNA stimulation studies were performed using RAW 264.7 mouse peritoneal macrophage cells (ATCC). The RAW macrophage cells were grown and maintained in Dulbecco’s Modified Eagle Media (DMEM) containing 4.5 g/L glucose, and supplemented with 10% fetal bovine serum and 1% penicillin–streptomycin. The cells were grown to near confluence in 12-well culture plates; and then for the DNA stimulation studies, fresh serum-free media was utilized. The RAW macrophage cells were incubated for 18 hours in media containing telomere-depleted cell-free DNA from the placental explants or DNA extracted from mouse placental tissue (with or without telomere depletion) at DNA concentrations of 5 microgram (μg) per milliliter (mL) of media. At the end of the incubation period, the media was collected and utilized to measure the concentration of IL6 released into the media using an enzyme linked immunosorbent assay (Mouse IL6 ELISA (BioLegend,)) according to the manufacturer’s recommendations.

### Statistical analysis

One-way analysis of variance (ANOVA), ANOVA on ranks, the Mann-Whitney Rank Sum test, paired or unpaired t-tests were performed where appropriate. When significant, the ANOVA results underwent multiple comparisons testing with the Dunn test. The results were presented as mean ± standard deviation. Differences were considered statistically significant when p ≤ 0.05.

## Results and discussion

**Fig 1:**
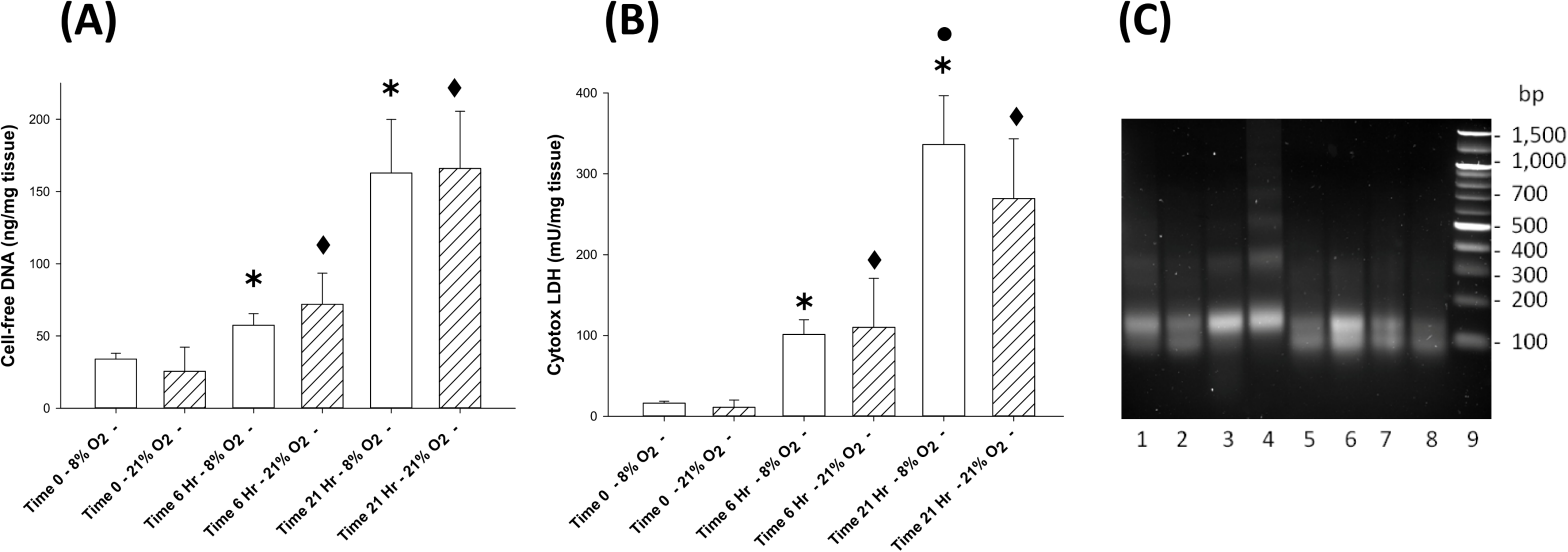
**A) Cell-free DNA response over time:** Bar graph demonstrating cell-free DNA (cfDNA) release by placental explants cultured in 8% O_2_ conditions (open bars) and in 21% O_2_ conditions (hatched bars) from time zero to 21 hours. Data reported in mean ± standard deviation (S.D.), N = 4–68, ✱ = p < 0.05 compared to time zero/8% O_2_ and ◆ = p < 0.05 compared to time zero/21% O_2_. **B) Cytotox LDH response over time:** Bar graph demonstrating LDH (by Cytotox assay) release by placental explants cultured in 8% O_2_ conditions (open bars) and in 21% O_2_ conditions (hatched bars) from time zero to 21 hours. Data reported in mean ± standard deviation (S.D.), N = 4–19, ✱ = p < 0.05 compared to time zero/8% O_2_, ◆ = p < 0.05 compared to time zero/21% O_2_ and ● = p < 0.05 compared to 21% O_2_ at 21 hours. **C) Cell-free DNA horizontal gel:** Image of a 1.5% agarose/TAE gel demonstrating cfDNA released by placental explants in 8% O_2_ (lanes 1, 2, 5, 6) and 21% O_2_ (lanes 3, 4, 7, 8) at 6 hours (lanes 1–4) and at 21 hours (lanes 5–8), and DNA size markers (lane 9). Gel stained with GelRed dye and transilluminated with UV light demonstrating cfDNA fragments of < 100 –< 400 base pair (bp) sizes. Each gel lane loaded with DNA extraction solution (containing 126 ng cfDNA for the 6 hour and 21 hour time points).

As observed in **Fig 1A**, under placental physiologic oxygen conditions (8% O_2_) the cfDNA release into the culture media by the placental explants at 6 hours increased by 69% above time zero; whereas by 21 hours, the cfDNA release increased 4.8-fold (both p < 0.05). In comparison, cfDNA released by placental explants cultured in ambient (placental hyperoxic) oxygen (21% O_2_) increased 2.8-fold at 6 hours and 6.5-fold at 21 hours (both p < 0.05). Although there was an interesting trend for the differences between cfDNA released in low O_2_ vs. ambient O_2_ conditions, none of the differences were statistically significant (**Fig 1A**). Of note, cfDNA released by the placental explants increased in parallel with the magnitude of cell death as demonstrated by the Cytotox LDH assay (**Fig 1B**); this observation was true for placental physiologic and ambient oxygen culture conditions. The cell-free DNA fragments released by mouse placental explants ranges in size from < 100 –< 400 bp (see **Fig 1C**); although in the 6 hour samples some faint DNA laddering (consistent with the ongoing apoptosis) can be observed in the 500–1,000 bp range.

**Fig 2:**
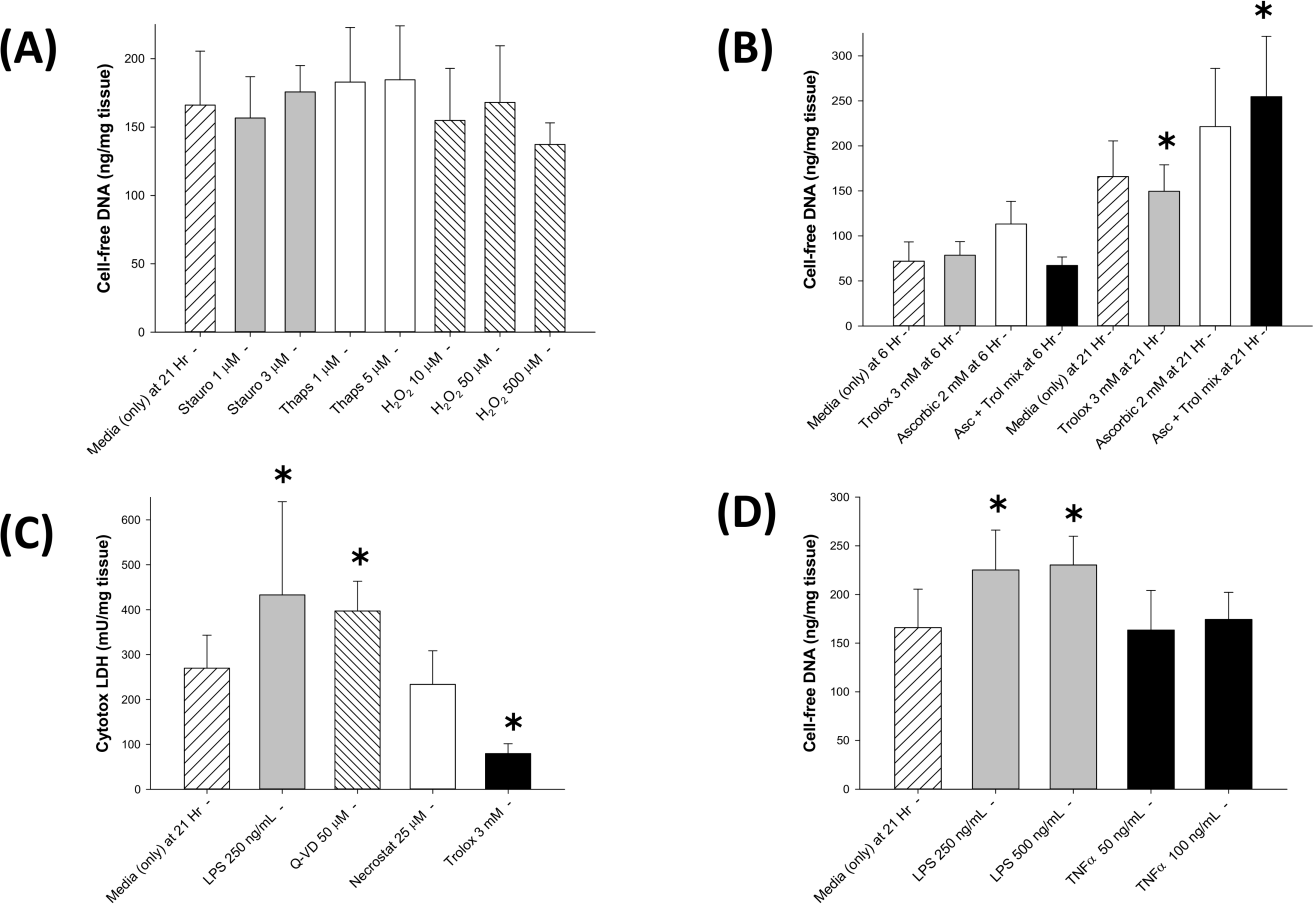
**A) Cell-free DNA response to staurosporine, thapsigargin & hydrogen peroxide:** Bar graph demonstrating cell-free DNA (cfDNA) release into the culture media by mouse placental explants cultured in 21% O_2_ conditions for 21 hours in the presence of tissue culture media alone (broad hatched bar), staurosporine (gray bars), thapsigargin (open bars), and hydrogen peroxide (H_2_O_2_, narrow hatched bars). Data reported in mean ± S.D., N = 4–57, and no significant differences for any bars compared to media alone. **B) Cell-free DNA response to ascorbic acid and trolox:** Bar graph demonstrating cell-free DNA (cfDNA) release into the culture media by mouse placental explants cultured in 21% O_2_ conditions at 6 hours and 21 hours in the presence of tissue culture media alone (hatched bars), trolox (Vitamin E analog, gray bars), ascorbic acid (Vitamin C, open bars) and a combination of trolox (1 mM) and ascorbic acid (2 mM) (Asc+Trol mix; black bars); data reported in mean ± S.D., N = 3–57, and ✱ = p < 0.05 compared to culture media alone at 21 hours. **C) Cytotox LDH response to LPS, Q-VD-OPh, necrostatin-1 & trolox:** Bar graphs demonstrating LDH (by Cytotox assay) release into the culture media by mouse placental explants cultured in 21% O_2_ conditions for 21 hours in the presence of tissue culture media alone (broad hatched bar), lipopolysaccharide (LPS, gray bar), Q-VD-OPh (narrow hatched bar), necrostatin-1 (Necrostat, open bar) and trolox (black bar). Data reported in mean ± S.D., N = 6–19, and ✱ = p < 0.05 compared to culture media alone. **D) Cell-free DNA response to LPS & TNFα:** Bar graph demonstrating cfDNA release into the culture media by mouse placental explants cultured in 21% O_2_ conditions for 21 hours in the presence of tissue culture media alone (hatched bar), LPS (gray bars) and tumor necrosis factor-α (TNFα, black bars). Data reported in mean ± S.D., N = 6–57, and ✱ = p < 0.05 compared to culture media alone.

For placental tissue, low (8%) oxygen levels have been described as best reflecting normal physiologic conditions; whereas, the ambient room air (21%) oxygen levels are high, resulting in oxidative stress [9]. As noted above, these studies demonstrate that the magnitude of cfDNA release and cell death are comparable at 6 and 21 hours with placental explants in physiologic (low O_2_) or ambient O_2_ (high O_2_) conditions. Therefore, the following studies were performed to determine if stimulators of apoptosis or enhanced oxidative stress further increased cfDNA release by the placental explants in ambient O_2_ conditions. As observed in **Fig 2A**, neither staurosporine nor thapsigargin increased the cfDNA release; interestingly, the same was true for H_2_O_2_ even at high concentrations. Oxidative stress can be mitigated by exogenous antioxidants including Vitamin C (ascorbic acid) and Vitamin E (α tocopherol) [6]. Studies performed with the addition of ascorbic acid alone or in combination with trolox (a water soluble Vitamin E analog) failed to decrease cfDNA release at 6 or 21 hours; and the combination actually mildly increased cfDNA release at 21 hours (**Fig 2B**). In contrast, trolox alone produced a modest, but statistically significant decreased in cfDNA release at 21 hours (**Fig 2B**). Interestingly, trolox markedly decreased cell death in these placental explants as demonstrated by an almost 80% decrease in LDH levels in the culture media (**Fig 2C**).

LPS has previously been reported to stimulate apoptosis in mouse placental tissue [16, 20]; therefore, placental explant studies were performed to determine if LPS also enhances cfDNA release. As observed in **Fig 2D**, LPS at 250 and 500 ng/mL produced a significant increase in cfDNA release. Studies assessing cell death confirmed that these two concentrations of LPS stimulated similar elevations of LDH levels at 21 hours; the significant increase in LDH concentration (p < 0.05) for the 250 ng/mL LPS treated placental explants is shown in **Fig 2C**. In contrast, TNFα (a proinflammatory cytokine) had no additive effect on cfDNA release by the placental explants at 6 hours or at 21 hours (**Fig 2D**).

**Fig 3:**
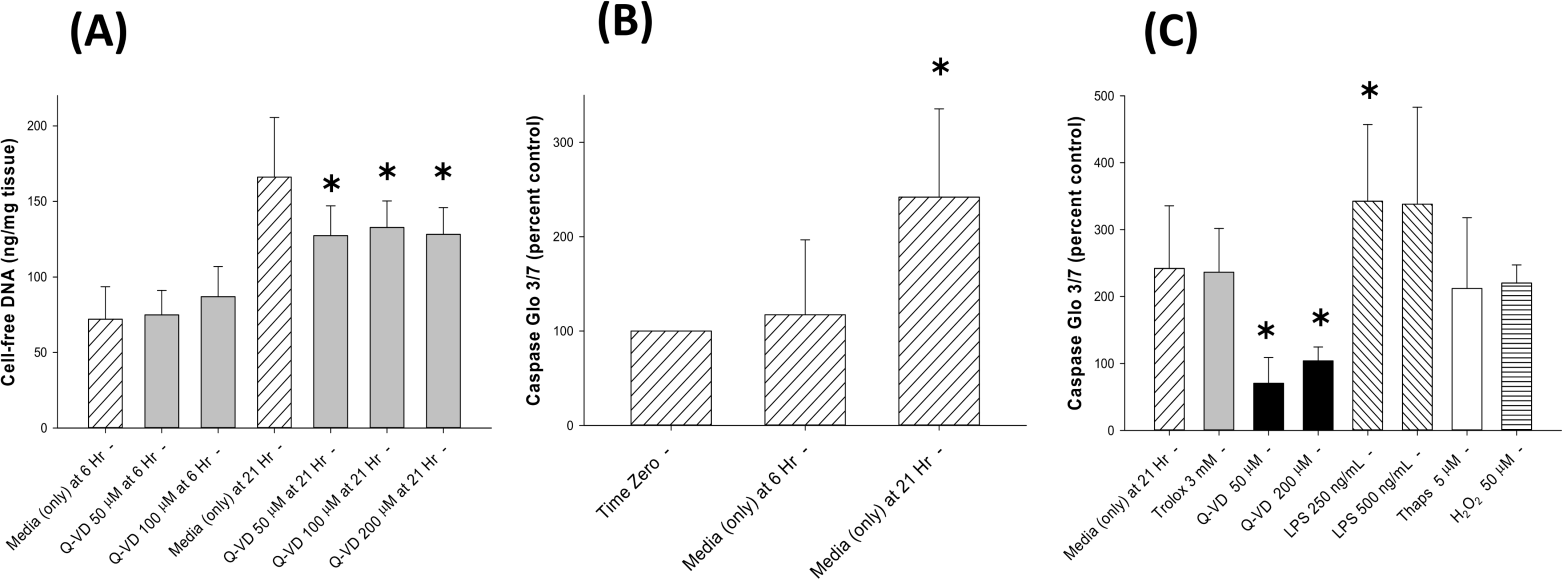
A) Cell-free DNA response to Q-VD-OPh: Bar graphs demonstrating cell-free DNA (cfDNA) release into the culture media by mouse placental explants cultured in 21% O_2_ conditions at 6 hours and 21 hours in the presence of tissue culture media alone (hatched bars) or Q-VD-OPh (a pan-caspase inhibitor, gray bars); data reported in mean ± S.D., N = 6–57, and ✱ = p < 0.05 compared to culture media alone at 21 hours. B) Caspase 3/7 response over time: Bar graph demonstrating caspase 3 and 7 activity (by Caspase Glo 3/7 assay) in tissue homogenates of placental explants cultured in 21% O_2_ conditions at 6 hours and 21 hours in the presence of tissue culture media alone; data reported in mean ± S.D., N = 5–12, and ✱ = p < 0.05 compared to time zero. C) Caspase 3/7 response to trolox, Q-VD-OPh, LPS, thapsigargin & hydrogen peroxide: Bar graph demonstrating caspase 3 and 7 activity in tissue homogenates of placental explants cultured in 21% O_2_ conditions at 21 hours in the presence of tissue culture media alone (broad hatched bars), trolox (gray bar), Q-VD-OPh (black bars), LPS (narrow hatched bars), thapsigargin (open bar) and hydrogen peroxide (H_2_O_2_, horizontal hatched bars); data reported in mean ± S.D., N = 4–12, and ✱ = p < 0.05 compared to culture media alone at 21 hours.

**Fig 4:**
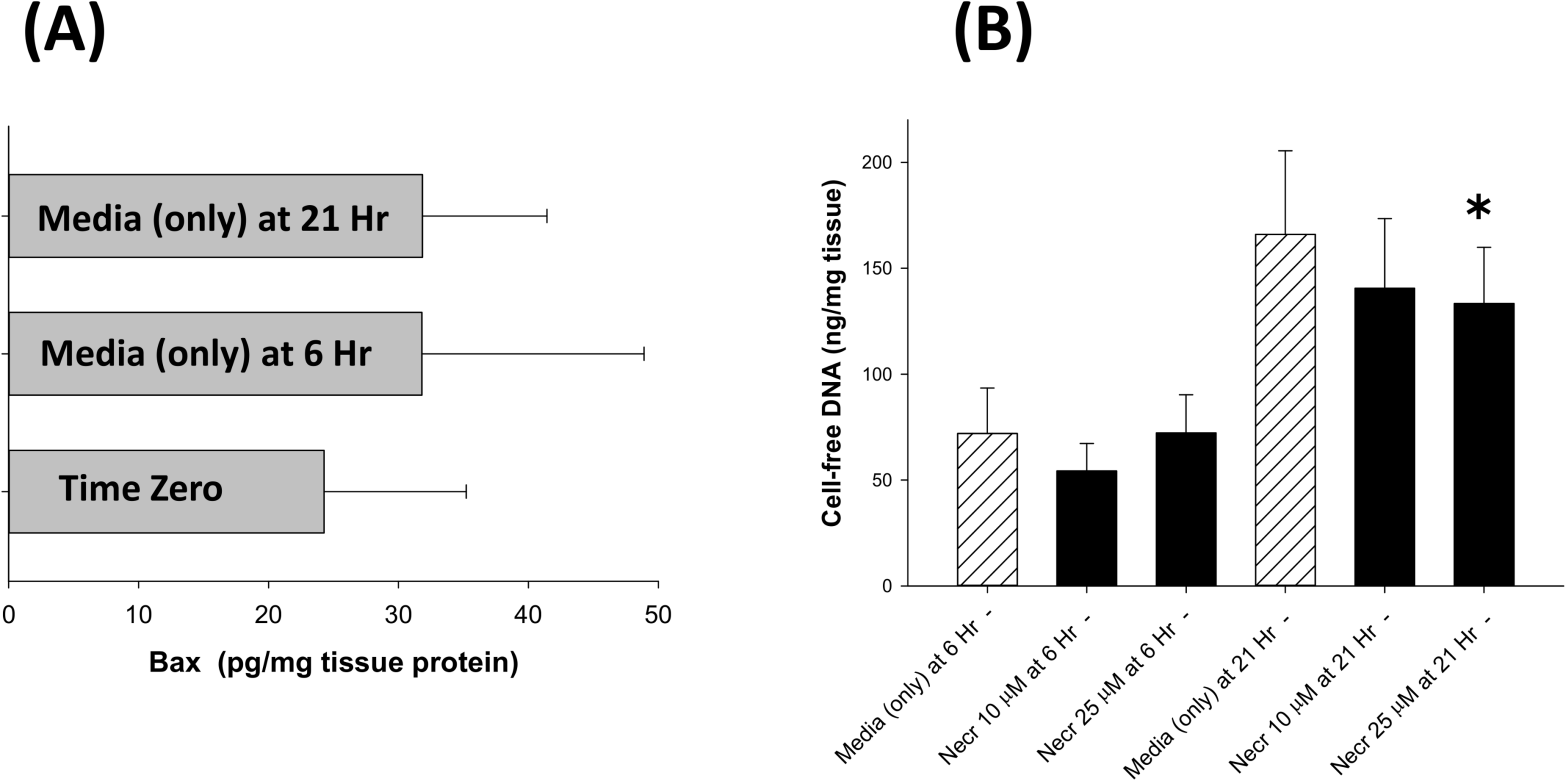
A) Bax expression in placental explants: Horizontal bar graph demonstrating expression of the pro-apoptotic Bax protein in homogenates of mouse placental explants cultured in 21% O_2_ conditions at time zero, 6 hours and 21 hours in the presence of tissue culture media alone. Bax expression normalized for tissue protein and reported as picograms (pg) per mg tissue protein; data reported in mean ± S.D., N = 16–21, and although a suggestive trend, the apparent increase in Bax expression was not statistically significant. B) Cell-free DNA response to necrostatin-1: Bar graph demonstrating cell-free DNA (cfDNA) release into the culture media by mouse placental explants cultured in 21% O_2_ conditions at 6 hours and 21 hours in the presence of tissue culture media alone (hatched bars) or necrostatin-1 (Necr; black bars); data reported in mean ± S.D., N = 6–57, and ✱ = p < 0.05 compared to culture media alone at 21 hours.

As mentioned previously, the release of cfDNA from the placental trophoblast cells is believed to involve cellular apoptosis and/or necrosis [1, 2]. To address these possibilities, the following mouse placental explant studies were performed. Q-VD-OPh (a pan-caspase inhibitor) produced a modest, but significant decrease in cfDNA release at 21 hours (p<0.05), as observed in **Fig 3A**. Interestingly, this pan-caspase inhibitor produced a modest increase in cell death as indicated by the LDH concentrations in the culture media (**Fig 2C**); apparently reflecting cell-death not mediated by apoptosis and not associated with cfDNA release. To further assess the role of caspase-induced apoptosis during the release of cfDNA, the levels of activated caspases-3 and -7 were determined in explant homogenates. As seen in **Fig 3B**, activated caspase 3/7 levels increased from time zero to 21 hours, an increase that paralleled the increase in cfDNA release. Activated caspase 3/7 levels were markedly decreased in the Q-VD-OPh treated placental explant cultures, and significantly increased in the LPS treated tissue (**Fig 3C**); whereas, the levels in the trolox, thapsigargin and H_2_O_2_ treated placental cultures were not significantly different from the untreated controls at 21 hours. Studies performed to assess the expression of the pro-apoptotic Bax protein in the placental explants demonstrated a trend toward increased expression at 6 and 21 hours (albeit not statistically significant); see **Fig 4A**.

Necroptosis is another pathway leading to programmed cell death; necroptosis is inhibited by necrostatin-1 which blocks RIP1 kinase, an essential component of the pathway [14, 15]. As observed in **Fig 4B**, necrostatin-1 had no significant effect on cfDNA release at 6 hours; however, using 25 μM, necrostatin-1 produced a modest decrease in cfDNA at 21 hours. In contrast, necrostatin-1 had no significant effect on overall cell death as shown in **Fig 2C**.

**Fig 5:**
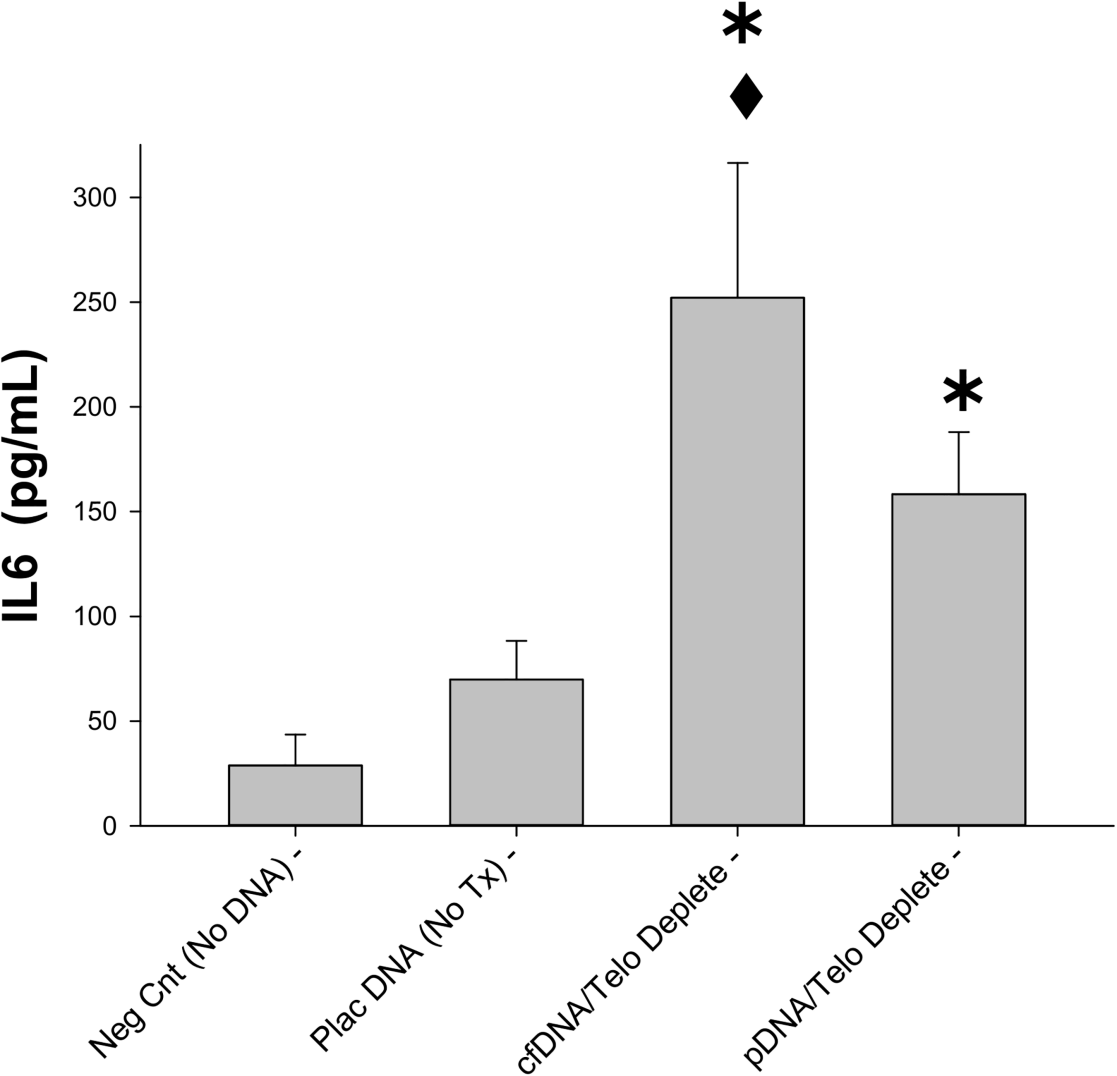
Stimulation of macrophage IL6 secretion. Bar graph demonstrating IL6 secretion (pg/mL culture media) by cultured RAW macrophage cells in response to stimulation by untreated placental DNA (Plac DNA (No Tx)), placental explant cell-free DNA depleted of telomere regions (cfDNA/Telo deplete), and placental tissue DNA depleted of telomere regions (pDNA/Telo deplete),. Negative control (Neg Cnt (No DNA)) = IL6 in media of unstimulated RAW cells. Data reported in mean ± S.D., N = 4–9, ✱ = p < 0.05 compared to untreated placental DNA and ◆ = p < 0.001 for cfDNA/Telo deplete compared to pDNA/Telo deplete.

In a recent report, we have demonstrated that placental DNA when complexed with DOTAP (a cationic liposome forming compound which facilitates intracellular DNA entry) and placental tissue DNA (depleted of the inhibitory telomere regions) both stimulate a significant increase in IL6 release by RAW macrophage cells; an effect that is mediated by TLR9 stimulation [18]. Based on these previous observations, the current study sought to determine if cfDNA from placental explants could also stimulate IL6 release by cultured macrophage cells. As observed in **Fig 5**, telomere-depleted cfDNA stimulated a significant increase in IL6 release by the RAW macrophage cells. Interestingly, the IL6 response to cfDNA was significantly higher than that produced by telomere-depleted placental DNA during these studies (p < 0.001).

In summary, these studies have confirmed the ability of mouse placental tissue to release cfDNA that is consistent in size with cell-free fetal DNA found in maternal plasma during pregnancy. These studies also demonstrated that cfDNA release was significantly increased over time and in the presence of LPS, and decreased by an antioxidant vitamin E analog. Whereas, during placental hyperoxic conditions, cfDNA release was not further enhanced by stimulators of apoptosis, or by additional oxidative stress produced by hydrogen peroxide. Supporting a possible role for caspase-mediated apoptosis, the amounts of cfDNA were significantly lower in cultures containing Q-VD-OPh, a pan-caspase inhibitor; and activated caspase 3/7 tissue levels increased in parallel with the rise in cfDNA levels in the explant culture media. These studies have demonstrated that increased cfDNA release by mouse placental tissue occurred in parallel with cell death that was associated with caspase-dependent mechanisms. These studies also demonstrated that cfDNA released by placental explants is functionally active in regard to its ability to stimulate IL6 secretion by mouse RAW peritoneal macrophage cells.

The presence of cell-free DNA from fetal sources in maternal plasma was first describe in 1997 by Lo and coinvestigators, noting that it accounts for 3.4–6.2% of the total cell-free fetal DNA present in maternal plasma, and that it has a short half-life averaging 16.3 minutes [21–23]. These investigators also found that cell-free fetal DNA fragment sizes begin at about 100 bp, with 80% of the fragments less than 193 bp in size, and none were greater than 313 bp [24]. These reported cell-free fetal DNA fragment sizes in maternal plasma are very consistent with the sizes of the cfDNA fragments we found to be released by mouse placental explants.

Lo et al. [22] also described an almost 12-fold rise in cell-free fetal DNA concentration by late gestation in women sampled sequentially throughout pregnancy. This observation has been confirmed by other investigators, including Ariga et al. [25], who observed a 13-fold increase in cell-free fetal DNA peaking at term; Birch et al. [26], who noted a log scale increase in cell-free fetal DNA concentration through gestation; and Wang et al. [27], who described a steady increase at a rate of 1 percent per week after 20 weeks of gestation, peaking at the end of gestation. In another published report, Majer et al. [28] described a significant inverse correlation for the days elapsed between blood collection and birth (i.e. the higher the cell-free fetal DNA level, the shorter the interval to delivery).

Maternal plasma cell-free fetal DNA derived from fetal/placental sources is not unique to human pregnancies. In pregnant mice, Khosrotehrani et al. [29] described an increase in cell-free fetal DNA levels during each of the 3 weeks of gestation. Wang et al. [30] reported the presence of cell-free fetal DNA in the plasma from pregnant cows, and de Leon et al. [31] made similar observations in pregnant horses. In pregnant sheep, Kadivar et al. [32] detected a 1.65-fold increase in maternal plasma cell-free fetal DNA levels for the last two months of gestation compared to the middle months. In studies using pregnant Rhesus monkeys (*Macaca mulatta*), Jimenez and Tarantal [33] observed a greater than 30-fold increase in cell-free fetal DNA levels between the first and third trimesters, with the greatest increase occurring during the last 30 days of gestation. In another Rhesus monkey study, Mitsunaga et al. [34] described a 5-fold increase in cell-free fetal DNA in maternal plasma during the last two weeks of gestation, peaking just before parturition and then falling to undetectable levels by 6 hours postpartum.

As discussed previously, without much direct evidence early studies suggested that cell-free fetal DNA is derived from trophoblast cells as they undergo normal turnover during cellular apoptosis and/or necrosis [1–5]. The studies by Tjoa et al. [6] were the first to directly observe cell-free DNA (cfDNA) release by placental tissue. Specifically, these investigators utilized human placental explant cultures to demonstrate the release of cfDNA which was enhanced in response to oxidative stress [6]. In addition, Tjoa et al. [6] observed that the amount of cfDNA released by the placental explants was mildly suppressed by the antioxidant vitamins, including trolox (a Vitamin E analog). Our studies in the present report have confirmed the ability of placental explants to release cfDNA into the media, an effect that was suppressed by trolox, but not by Vitamin C. In addition, we observed that cfDNA release by the mouse placental explants was associated with apoptosis, as demonstrated by the reduction with the pan-caspase inhibitor (Q-VD-OPh) and the enhancement by LPS (which also significantly increased cell death in this tissue). Interestingly, placental explants cultured in 8% O_2_ (conditions thought to replicate normal placental physiology [9, 35]) produced no statistically significant different effects on cfDNA release at 6 hours when compared to explants cultured in 21% O_2_; and the same was true for cultures under these two different O_2_ conditions at 21 hours. Our results in regard to no difference in cell death between low oxygen and ambient oxygen conditions are similar to the results reported by Depoix et al. [35]. These investigators cultured cytotrophoblast cells isolated from human placentas at term under hypoxic (2.5% O_2_) vs. ambient (21% O_2_) conditions, and found a similar 45% decrease in cell viability at 24 hours with both culture conditions, along with no difference between these two oxygen conditions for caspase 3/7, caspase-8 and caspase 9 activity [35].

## Conclusion

As noted previously, the possibility that cell-free fetal DNA released by the placenta and/or fetal membranes serves as a trigger for parturition through its ability to stimulate an innate immune response has recently been proposed [7, 8]. Such a response would result in the intrauterine production of the proinflammatory cytokines and chemokines that have been shown to lead to cervical ripening, rupture of the membranes and the onset of phasic myometrial contractions [7, 8]. The current studies have confirmed the ability of placental tissue to release significant amounts of cfDNA, a phenomenon that appears to be mediated, at least in part, by apoptosis of cells within the placenta. Our current studies have also demonstrated that cfDNA released by the placental explants is able to stimulate a proinflammatory response as demonstrated by increased IL6 release by the macrophage cells. In summary, these studies have provided support for the novel hypothesis that cell-free fetal DNA released by placental tissue potentially plays a mechanistically important role during the events leading to the spontaneous onset of parturition.
